# Proton Pump Inhibitors and Disproportionate Reporting of Acute Kidney Injury and Tubulointerstitial Nephritis: A FAERS Pharmacovigilance Study, 2020–2025

**DOI:** 10.3390/jcm15031298

**Published:** 2026-02-06

**Authors:** Thamir M. Alshammari, Mohammad Kanan Alshammari, Hind M. Alosaimi, Ayesha Yasmeen, Mamoon H. Syed

**Affiliations:** 1 Department of Clinical Practice, College of Pharmacy, Jazan University, Jazan 45142, Saudi Arabia; amoinuddin@jazanu.edu.sa (A.Y.); mhsyed@jazanu.edu.sa (M.H.S.); 2 Pharmacy Practice Research Unit, College of Pharmacy, Jazan University, Jazan 45142, Saudi Arabia; 3 Department of Pharmaceutical Care Administration, Research and Studies Unit at Rafha General Hospital, Northern Border Healthcare Cluster, Rafha 76312, Saudi Arabia; mokaalshammari@moh.gov.sa; 4 Department of Pharmacy Services Administration, King Fahad Medical City, Riyadh Second Health Cluster, Riyadh 12211, Saudi Arabia; halosaimi@kfmc.med.sa

**Keywords:** acute kidney injury, disproportionality analysis, pharmacovigilance, proton pump inhibitors, tubulointerstitial nephritis

## Abstract

**Background/Objectives:** Proton pump inhibitors (PPIs) are widely used, yet questions persist about kidney-related adverse events. We evaluated disproportional reporting of acute kidney injury (AKI) and tubulointerstitial nephritis (TIN) with PPIs in the FDA Adverse Event Reporting System (FAERS) from 2020 to 2025. **Methods:** FAERS reports were screened using MedDRA Preferred Terms. Report characteristics and annual counts of AKI and TIN reports were summarized. Reporting Odds Ratio (ROR), Proportional Reporting Ratio (PRR), Empirical Bayes Geometric Mean (EBGM), and Information Content (IC) were used to assess disproportionality. **Results:** We identified 13,654 PPI-associated AKI reports and 2409 TIN reports in FAERS (2020–2025). Reports were predominantly from the United States, and missing age/sex information was common. Hospitalization was reported in 12.3% of AKI and 22.7% of TIN reports, and death in 9.1% and 5.0%, respectively. Across all years, disproportionality analyses using ROR, PRR, EBGM, and IC consistently met signal thresholds for both outcomes, with stronger signals in 2020–2022 and attenuation thereafter alongside declining report counts. **Conclusions:** FAERS data show persistent disproportional reporting of AKI and TIN with PPI use. Causality cannot be inferred, but the findings support cautious, indication-based PPI prescribing and highlight the need for robust studies to clarify renal safety.

## 1. Introduction

Proton pump inhibitors (PPIs) are widely used for the treatment of acid-related gastrointestinal disorders, including peptic ulcer disease, gastroesophageal reflux disease, and *Helicobacter pylori* infection [1]. Since their introduction in the late 1980s, their potent and sustained acid-suppressing effect, convenient dosing schedules, and favorable short-term safety profile have led to extensive prescribing and frequent over-the-counter use in many settings [2]. However, concerns have been raised about the safety of long-term (chronic) PPI therapy, particularly when continued beyond the initial treatment course or without a clear ongoing indication [1,2]. A recent review has suggested that extended-duration PPI use may be associated with adverse health effects, including potential kidney-related outcomes, although many patients appear to tolerate these medications without clinically apparent problems [1,3]. Nevertheless, observational studies and case reports have reported potential associations between PPI exposure and kidney outcomes, particularly acute kidney injury (AKI) and tubulointerstitial nephritis (TIN) [4,5]; however, these designs are hypothesis-generating and cannot establish causality. AKI is characterized by an abrupt decline in kidney function that may be reversible but can progress to chronic kidney disease if it is not recognized and managed promptly [6]. TIN involves inflammation of the renal tubules and interstitium and may manifest as acute or chronic impairment in kidney function [5]. The biological mechanisms by which PPIs may contribute to kidney injury remain incompletely understood, with proposed pathways such as idiosyncratic immune-mediated TIN, alterations in tubular transport, and microvascular injury [6]. Evidence remains insufficient to establish a causal relationship between PPIs and kidney injury, as some studies report an association, while residual confounding and other biases limit causal interpretation [7]. Variability in study design and population characteristics has further complicated efforts to clarify the renal safety profile of PPIs [8].

Given the ongoing uncertainty around the renal safety of PPIs, there is still a need for robust large-scale studies to clarify the potential kidney risks associated with these widely used medications [7]. The present study seeks to address this gap by using disproportionality analysis to explore the association between PPI use and reports of AKI and TIN in a large pharmacovigilance database. Disproportionality analysis is widely used to identify drug-event pairs that are reported more frequently than expected, based on drug utilization patterns and adverse event reporting [9,10]. Applying this approach to a large pharmacovigilance dataset allows for a systematic assessment of potential renal risks associated with PPI use. The resulting signals from such analyses can aid clinicians and shape public health recommendations around long-term use of PPIs. Identification of strong and consistent renal safety signals may prompt reevaluation of prescribing practices, especially for patients requiring long-term or high-dose PPI therapy. In contrast, reassuring safety signals would support appropriate PPI use within current indications and provide greater confidence to clinicians and patients. Here, ‘signal detection’ refers to disproportionality-based screening within FAERS and is hypothesis-generating rather than risk- or causality-estimating [10]. Therefore, this study aimed to conduct disproportionality-based signal detection in the U.S. Food and Drug Administration (FDA) Adverse Event Reporting System (FAERS) to characterize reporting patterns for AKI and TIN associated with PPIs and to describe the frequency of PPI-associated AKI and TIN reports submitted between 2020 and 2025.

## 2. Materials and Methods

### 2.1. Data Source

This pharmacovigilance study utilized data from the FAERS public dashboard [11] to identify and retrieve reports of PPI-associated AKI and TIN submitted between 2020 and 2025. Globally, the FAERS database receives spontaneous reports of suspected adverse events associated with drugs and biologics through post-marketing surveillance activities from healthcare professionals, manufacturers, and patients or consumers [11]. The Medical Dictionary for Regulatory Activities (MedDRA) is used to code each adverse event in the FAERS database and assigns a unique ID for consistency and uniformity [12]. This study adhered to the READUS-PV (REporting of A Disproportionality analysis for drUg Safety signal detection using individual case safety reports in PharmacoVigilance) guidelines for reporting disproportionality-based signal detection analyses using spontaneous reporting databases such as FAERS [13,14].

### 2.2. Data Retrieval

All reports of AKI and TIN following PPI use were extracted from the FAERS database for the period 2020 to 2025. To capture these events, we used the search terms “acute kidney inj” and “tubulointers”, and we included the MedDRA Preferred Terms “Acute Kidney Injury” and “Tubulointerstitial Nephritis” to characterize the renal safety profile of PPIs during the study period. Patient demographics and clinical characteristics such as age, sex, country from which reports were submitted, reporter type, and reported outcomes, along with the yearly frequency of AKI and TIN cases reported from 2020 to 2025, were obtained from the FAERS database for analysis. These variables were included in the analysis to describe the profile of PPI-associated AKI and TIN reports.

### 2.3. Disproportionality Analysis

For disproportionality analysis and pharmacovigilance signal detection (disproportionality-based screening within FAERS), the Reporting Odds Ratio (ROR), Proportional Reporting Ratio (PRR), Empirical Bayes Geometric Mean (EBGM), and the Information Component (IC) were used as data-mining algorithms [9,15]. PPI exposure was defined as any report in which at least one PPI was listed as a suspect drug. Reports listing PPIs only as concomitant medications were excluded from the primary analysis. For each outcome (AKI and TIN) and each calendar year, 2 × 2 contingency tables were made with the following cells: (a) reports of the outcome with PPIs, (b) reports of other events with PPIs, (c) reports of the outcome with all other drugs, and (d) reports of other events with all other drugs. Disproportionality signals were considered present when the lower bound of the 95% confidence interval for the ROR exceeded 1 with at least three cases and when PRR was ≥2 with χ^2^ ≥ 4. For the Bayesian measures, EB05 > 2 and IC025 > 0 were taken as indicative of a signal [16].

### 2.4. Statistical Analysis

Descriptive analyses were performed for all demographic and clinical variables included in the study. Frequencies and percentages were calculated for each variable and are presented to summarize the distribution of PPI-associated AKI and TIN reports. Both AKI and TIN cases were stratified by reporting year from 2020 to 2025. Missing or uncoded values for demographic variables (e.g., age, sex), country, and reporter type were retained and reported as ‘Not Specified’; no imputation was performed. All statistical analyses were performed by using IBM SPSS Statistics, version 23 (IBM Corp., Armonk, NY, USA).

## 3. Results

We identified 13,654 reports of AKI and 2409 reports of TIN-associated PPI use in FAERS between 2020 and 2025. Age and sex distributions are summarized in Table 1. Among cases with recorded age, the most frequent age group was 60–<90 years, with 24.4% of AKI and 34.4% of TIN reports. However, age was missing for 48.6% of AKI and 32.4% of TIN cases. Females were the largest identified group in both AKI and TIN (35.3% and 44.5%, respectively), although sex was not specified in 36.2% of AKI and 22.6% of TIN reports. Reports with missing age or sex were not excluded and are presented as ‘Not Specified’ in Table 1; this missingness reflects incomplete field capture in FAERS and limits interpretation of subgroup distributions.

Disproportionality analysis of AKI reports associated with PPIs from 2020 to 2025 using four data mining algorithms (ROR, PRR, EBGM, and IC) is presented in Table 2. Findings revealed that each year, the reported cases of AKI showed strong disproportionality signals with PPIs in every year, with the strongest signals in 2021, with an ROR of 52.5 and PRR of 38.02, followed by 2022. Whereas a gradual decline in signal strength was observed from 2023 onwards, with the lowest values recorded in 2024 and 2025. This indicates a decreasing trend in reported disproportionality between PPIs and AKI over the years, which may be attributed to the smaller number of reports in these years.

A comparable pattern was seen for TIN (Table 3). TIN reports exhibited very strong disproportionality signals with PPIs in all years, with the highest ROR and PRR values in 2020–2022 (maximum ROR 81.10; PRR 77.64 in 2020) and progressive attenuation thereafter (ROR 36.16 and PRR 35.19 in 2023; ROR 27.12 and PRR 26.57 in 2024; ROR 23.65 and PRR 23.21 in 2025). EBGM and IC values also remained above signal thresholds in every year, confirming persistent but gradually attenuating disproportional reporting of TIN with PPI use over the study period. Across 2020–2025, agent-stratified descriptive summaries of AKI and TIN reporting by individual PPI are provided in Supplementary Tables S1 and S2. AKI reporting composition ranged from 0.0% to 37.22% (highest observed: esomeprazole, 2021; 1826/4905), and TIN reporting composition ranged from 0.0% to 6.12% (highest observed: rabeprazole, 2020; 12/196). These summaries describe report composition within FAERS and should not be interpreted as incidence or comparative clinical risk.

**Figure 1:**
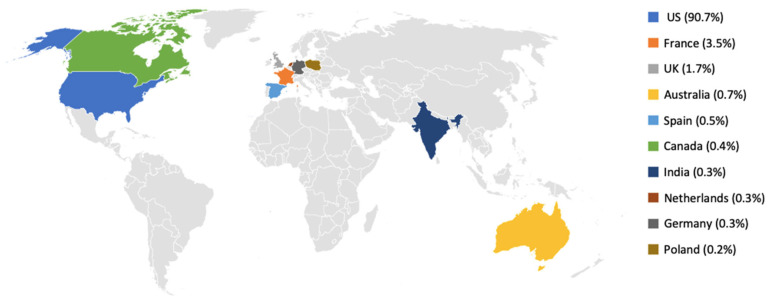
Geographical distribution of FAERS reports of PPI-associated AKI, 2020–2025. Gray indicates no reports from these countries or missing/uncoded country information. Percentages shown correspond to the top 10 reporting countries and therefore do not sum to 100%.

**Figure 2:**
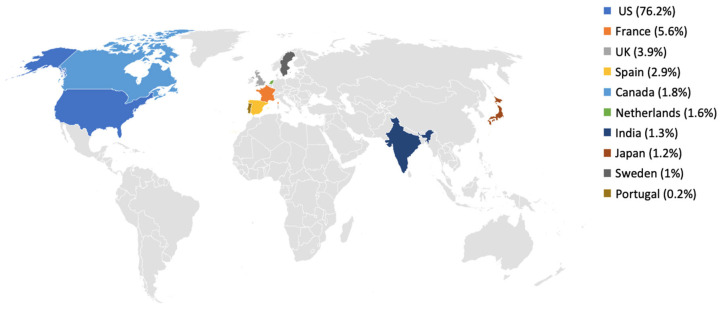
Geographical distribution of FAERS reports of PPI-associated TIN, 2020–2025. Gray indicates no reports from these countries or missing/uncoded country information. Percentages shown correspond to the top 10 reporting countries and therefore do not sum to 100%.

The geographical distribution of reports for AKI and TIN is presented in Figure 1 and Figure 2. For AKI, most reports originated from the United States (12,384/13,654; 90.7%), followed by France (3.5%) and the United Kingdom (1.7%), with each of the remaining countries contributing less than 1% of cases (Figure 1). A similar pattern was observed for TIN, where 1836/2409 reports (76.2%) originated from the United States, followed by France (5.6%) and the United Kingdom (3.9%), while other countries such as Spain, Canada, the Netherlands, India, Japan, Sweden and Portugal each contributed less than 3% of reports (Figure 2). Because FAERS captures reports submitted to the US FDA and the country field may be incomplete, the apparent absence or low frequency of reports from some regions (including Gulf countries) should be interpreted cautiously. In Figure 1 and Figure 2, gray areas indicate countries with no mappable country information in the FAERS extract and/or zero reports in the analyzed subset.

Reporter occupation is summarized in Table 4. Among AKI reports, the largest identified professional group was nurses (33.9%), followed by consumers (15.1%) and physicians (7.5%), while 35.5% of reports lacked coding for reporter occupation. For TIN, nurses again constituted the largest identified group (25.0%), followed by physicians (15.7%) and other healthcare professionals (14.6%). Pharmacists represented a small fraction of reports for both AKI (1.7%) and TIN (3.9%).

Clinical outcomes are shown in Table 5. In PPI-associated AKI reports, most outcomes were coded as “other serious” (10,332/13,654; 75.7%), with hospitalization reported in 1685 cases (12.3%) and death in 1240 cases (9.1%). For TIN, “other serious” outcomes were reported in 1600/2409 cases (66.4%), hospitalization in 546 cases (22.7%) and death in 120 cases (5.0%). As expected in a spontaneous reporting system enriched for serious events, most reports were classified as other serious outcomes rather than explicitly coded as hospitalization or death.

**Figure 3:**
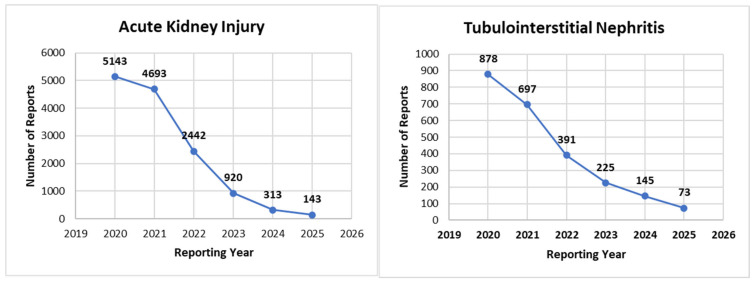
Yearly frequency of PPI-associated AKI and TIN reports in FAERS, 2020–2025.

Temporal trends in reporting are illustrated in Figure 3. For AKI, the highest number of reports following PPI use was observed in 2020 (5143/13,654; 37.7%), followed by 2021 (4693/13,654; 34.4%), with a progressive decline thereafter to 2442 reports (17.9%) in 2022, 920 (6.7%) in 2023, 313 (2.3%) in 2024 and 143 (1.0%) in 2025. A similar pattern was observed for TIN, with the greatest number of reports in 2020 (878/2409; 36.4%) and 2021 (697/2409; 28.9%), followed by 391 (16.2%) in 2022, 225 (9.3%) in 2023, 145 (6.0%) in 2024 and 73 (3.0%) in 2025. These patterns show a clear decline in the number of AKI and TIN reports involving PPIs over the study period.

## 4. Discussion

This study evaluated FAERS reports submitted between 2020 and 2025 and identified consistent disproportionality-based pharmacovigilance signals for AKI and TIN associated with PPIs. Signal estimates were higher in earlier years and attenuated in later years, a pattern that may reflect time-varying reporting behavior and changes in clinical practice rather than changes in absolute clinical risk. As with other spontaneous reporting systems, disproportionality estimates may be influenced by stimulated reporting, notoriety bias, and time-dependent reporting phenomena (e.g., Weber-type effects), particularly following heightened attention to specific adverse events [10,17,18]. Accordingly, these signals should be interpreted cautiously given that PPI use is often concentrated in clinical scenarios with intrinsically elevated AKI risk (hospitalization, critical illness, sepsis, and prophylactic co-prescribing), which cannot be adequately accounted for in FAERS [10,19]. Although agent-stratified summaries show variability, these patterns likely reflect differences in utilization, channeling, and time-varying reporting dynamics (and small denominators in certain strata) and should not be interpreted as evidence that one PPI is safer than another [10,19].

Among cases with recorded age in this study, patients aged 60 to <90 years constituted the largest group for both AKI and TIN. Among reports with recorded sex, a higher proportion of TIN reports were submitted for females; however, sex was missing for a substantial fraction of FAERS reports. While this missingness does not affect the primary disproportionality-based signal detection analyses, it limits the interpretability of sex-stratified descriptive patterns and precludes firm conclusions regarding sex-related susceptibility. Prior observational evidence has linked PPI exposure to adverse kidney outcomes and indicates that kidney risk is often higher in older adults across clinical settings, which is broadly consistent with the age distribution observed here [4]. In addition, observational evidence has linked PPI exposure and potentially non-indicated initiation to adverse longer-term kidney outcomes, including chronic kidney disease [20,21]. Nevertheless, sex-specific comparisons should be interpreted cautiously given differences in design and data completeness, and because AKI risk and outcomes vary by age and sex across clinical settings [22].

One plausible contributor to the higher signal estimates in earlier years is evolving prescribing practice, including increased emphasis on reviewing long-term therapy and deprescribing or stepping down treatment when indications are unclear [23]. In addition, trends in spontaneous reports should be interpreted cautiously because reporting behavior can vary over time, influencing both submitted case volumes and disproportionality estimates [17]. From a clinical standpoint, a biologically plausible pathway linking PPI exposure to acute renal injury is idiosyncratic immune-mediated tubulointerstitial nephritis, which may present clinically as AKI and can be under-recognized when creatinine changes are attributed to acute illness or competing causes. However, because FAERS does not provide systematic information on indication, baseline renal function, illness severity, or time-aligned co-medications, the observed signals may also reflect clinical context and co-exposure to nephrotoxic or renal stressor therapies rather than a direct drug effect. During 2020–2022, the COVID-19 pandemic may have altered healthcare utilization and prescribing patterns, concentrating PPI exposure in hospitalized and critically ill patients and increasing co-therapy (e.g., systemic corticosteroids and antithrombotics) where gastroprotection is frequently used. Such pandemic-era shifts may have contributed to higher renal adverse event reporting volumes and inflated disproportionality estimates through reporting dynamics and clinical context rather than reflecting a change in drug-specific causal risk [24]. Renal injury may also occur as a complication of COVID-19 itself [25]. In addition, underreporting remains a well-recognized limitation of spontaneous reporting systems, and it can vary by setting and by reporter, which may further affect observed trends [26,27].

The geographical distribution of reports showed that most reports originated from the United States, which accounted for 90.7% of AKI reports and 76.2% of TIN reports. This pattern may partly reflect the larger population size and higher PPI utilization in the US, but it may also reflect differences in adverse event reporting frequency and reporting processes between countries [10,19]. In addition, underrepresentation from some regions may occur because FAERS is FDA-centric and relies on submissions to the FDA, so geographic gaps should not be interpreted as evidence of lower incidence [10]. In line with this, variability in regulatory requirements and case reporting standards across regions has been documented, highlighting that differences in regulatory requirements and reporting standards may contribute to geographic variability. [28].

The variation in reporting patterns across reporter groups was also noteworthy. Nurses contributed the largest share of AKI and TIN reports, followed by consumers for AKI and physicians for TIN. The large contribution from nurses is notable and is consistent with evidence that nursing staff can play a meaningful role in recognizing and submitting suspected adverse drug reaction reports within spontaneous reporting systems [29]. The sizeable proportion of consumer reports also aligns with evidence that direct patient reporting adds useful safety information to pharmacovigilance databases and can complement reports from healthcare professionals [30].

With respect to outcomes, most AKI and TIN reports were categorized as “other,” limiting our capacity to accurately characterize the severity of these adverse events. FAERS reports do not always include laboratory values (e.g., serum creatinine) or longitudinal clinical course information, as these data are not mandatory for submission. Consequently, AKI reports may span a wide severity spectrum, from transient creatinine elevations to kidney failure requiring intervention, and we could not stage AKI or assess reversibility. Similarly, TIN in FAERS represents a coded diagnostic label that may reflect clinical suspicion rather than biopsy-confirmed disease. As a result, agent-level clinical extrapolation regarding severity, reversibility, or progression to chronic kidney disease is not possible from these reports. Nevertheless, the proportion of cases resulting in hospitalization (12.3% for AKI and 22.7% for TIN) and death (9.1% for AKI and 5% for TIN) is notable and underscores the potential seriousness of reported renal events in which PPIs were listed as suspect drugs. Observational evidence in CKD populations has also suggested an association between PPI exposure and higher risks of AKI and mortality, although such designs remain susceptible to confounding [31].

This study showed a steady decrease in reports of PPI-associated AKI and TIN between 2020 and 2025. On face value, this decline could reflect closer monitoring and more careful prescribing over time. However, other explanations should also be considered, including reporting fatigue and broader shifts in reporting behavior or reporting processes within spontaneous reporting systems [24]. Time-dependent reporting patterns may also contribute to early peaks followed by lower reporting volumes in later years, a phenomenon often discussed in pharmacovigilance literature, although it is not consistently observed across medicines [18].

Our findings add to the current evidence on the renal effects of PPIs and reinforce the need for careful prescribing, particularly in higher-risk groups such as older adults and patients with other risk factors for kidney disease. Further research is required to explore whether the downward trend in reported cases reflects real improvements in patient safety or simply changes in reporting behavior. To get an accurate assessment of the risks, future research should focus on robust clinical and pharmacoepidemiologic studies with clearly defined comparison groups, and careful control for confounding will be essential to more accurately quantify any kidney risks associated with PPI use. In addition, enhancing the reliability and accuracy of adverse event reports, especially with respect to patient demographics, comorbidities, and outcomes, would further strengthen data, which would be more valuable for guiding clinical decisions and shaping regulatory policy.

Overall, the analysis of FAERS reports from 2020 to 2025 shows a persistent disproportionality signal for AKI and TIN in association with PPI use. Although the overall trend in reporting shows fewer submitted AKI and TIN reports over time, this should not be interpreted as evidence of improved safety, as spontaneous reporting is influenced by reporting behavior and utilization patterns. The continued presence of strong signals across several data mining algorithms emphasizes the need to balance the benefits and potential renal risks of PPI therapy, particularly in vulnerable groups.

### Limitation of the Study

It is essential to acknowledge the limitations inherent to this type of study, even though it provides useful insights. Firstly, there is a substantial risk of bias and underreporting in the FAERS database, which relies on spontaneous cases and may be subject to selective and time-varying reporting; therefore, apparent time trends may reflect reporting dynamics rather than changes in true clinical risk [17]. Secondly, because the database does not include detailed exposure information or a clearly defined control group, it is not possible to draw clear cause-and-effect conclusions or estimate true incidence rates. In addition, FAERS lacks denominator data and standardized information on dose, duration, adherence, and time-to-onset; reports frequently include multiple suspect and concomitant drugs without standardized attribution to a single product; and overall report completeness and clinical detail are variable [10]. Thirdly, the signals identified in this study should be regarded as hypothesis-generating. Confounding by indication and clinical context is particularly important for PPIs. In routine practice, PPIs are often initiated in hospitalized or critically ill patients for stress ulcer prophylaxis or gastrointestinal bleeding prophylaxis and in patients with advanced age and multimorbidity. These settings are independently associated with AKI risk due to acute illness, sepsis, hemodynamic instability, and frequent co-exposure to nephrotoxic or renal stressor therapies (e.g., NSAIDs, antithrombotics, diuretics, ACE inhibitors/ARBs, iodinated contrast, vancomycin, aminoglycosides, and vasopressors). Because FAERS does not provide systematic information on indication, illness severity, baseline renal function, or timing of co-medications, the observed disproportional reporting may be inflated by these factors rather than reflecting a direct drug effect. Accordingly, residual confounding cannot be excluded. Fourthly, our reliance on MedDRA Preferred Terms may have led to misclassification or incomplete capture of renal events coded under different kidney-related terms. In addition, MedDRA Preferred Terms and FAERS outcome codes do not capture AKI stage, creatinine trajectories, renal replacement therapy, or recovery, and they provide no reliable information on biopsy confirmation for TIN or longer-term progression to chronic kidney disease. Therefore, the clinical severity and diagnostic certainty of reported AKI/TIN events cannot be determined from FAERS. Potential duplication of reports, differences in how cases are verified, and variation in diagnostic criteria across settings may also have affected the observed patterns. Given the spontaneous, report-based nature of FAERS and the absence of denominator data and a defined comparator group, causality cannot be inferred from this study. In addition, definitive statements about demographic risk factors are limited because key variables (e.g., age and sex) were frequently missing. These limitations mean that our findings should be interpreted with caution and considered complementary rather than a replacement for evidence from well-controlled observational and interventional studies.

## 5. Conclusions

This FAERS-based pharmacovigilance study identified persistent disproportional reporting signals for AKI and TIN in association with PPI use between 2020 and 2025, with signals highest in the earlier years but present throughout the study period. These findings are hypothesis-generating and do not establish causality or quantify incidence. Nevertheless, the persistence of signals across multiple data-mining algorithms and the presence of serious reported outcomes support careful, indication-focused PPI use, particularly in older adults and other vulnerable patients. Periodic review of long-term PPI therapy and deprescribing when there is no clear ongoing indication may be considered. Well-designed clinical and pharmacoepidemiologic studies with appropriate comparators and confounding control are needed to more accurately quantify renal risks associated with PPI use.

## Figures and Tables

**Table 1:** Characteristics of PPI-associated AKI and TIN reports in FAERS, 2020–2025.

Age	Acute Kidney Injury		Tubulointerstitial Nephritis	
	n	%	n	%
0–<18 years	36	0.3	30	1.2
18–<40 years	476	3.5	161	6.7
40–<60 years	3097	22.7	597	24.8
60–<90 years	3335	24.4	829	34.4
≥90 years	76	0.6	11	0.5
Not Specified	6634	48.6	781	32.4
Total	13,654	100.0	2409	100.0
**Gender**				
Female	4825	35.3	1072	44.5
Male	3885	28.5	792	32.9
Not Specified	4944	36.2	545	22.6
Total	13,654	100.0	2409	100.0

Note: Percentages may not sum to exactly 100% due to rounding.

**Table 2:** Disproportionality analysis of PPI-associated AKI in FAERS, 2020–2025.

Year	Number of AKI Events with PPIs	Reported ADEs with PPIs	ROR	PRR	EBGM	IC
			(95% CI)	(95% CI)		
2020	5143	20,333	35.04 (33.82–36.29)	26.42 (25.51–27.34)	20.38	4.34
2021	4693	16,690	52.50 (50.56–54.52)	38.02 (36.61–39.48)	28.45	4.83
2022	2442	11,116	40.00 (38.12–41.98)	31.43 (29.95–32.99)	26.38	4.72
2023	920	8113	16.17 (15.06–17.35)	14.45 (13.46–15.51)	13.47	3.75
2024	313	6870	5.78 (5.15–6.48)	5.56 (4.90–6.23)	5.41	2.43
2025 ^a^	143	3735	5.17 (4.36–6.12)	5.01 (4.23–5.93)	4.85	2.27

^a^ Data till June 2025; PPI: Proton Pump Inhibitor: AKI: Acute Kidney Injury; ADE: Adverse Drug Events; ROR: Reporting Odds Ratio; PRR: Proportional Reporting Ratio; EBGM: Empirical Bayes Geometrical Mean; IC: Information Content.

**Table 3:** Disproportionality analysis of PPI-associated TIN in FAERS, 2020–2025.

Year	Number of TIN Events with PPIs	Reported ADEs with PPIs	ROR	PRR	EBGM	IC
			(95% CI)	(95% CI)		
2020	878	20,333	81.10 (73.94–88.96)	77.64 (70.78–85.16)	40.23	5.33
2021	697	16,690	61.71 (56.21–67.74)	59.17 (53.90–64.96)	37.85	5.24
2022	391	11,116	61.80 (54.95–69.49)	59.62 (53.05–67.09)	41.67	5.38
2023	225	8113	36.16 (31.34–41.73)	35.19 (30.49–40.60)	28.35	4.82
2024	145	6870	27.12 (22.80–32.26)	26.57 (22.34–31.60)	22.21	4.47
2025 ^a^	73	3735	23.65 (18.53–30.18)	23.21 (18.18–29.62)	18.49	4.2

^a^ Data till June 2025; PPI: Proton Pump Inhibitor; TIN: Tubulointerstitial Nephritis; ADE: Adverse Drug Events; ROR: Reporting Odds Ratio; PRR: Proportional Reporting Ratio; EBGM: Empirical Bayes Geometrical Mean; IC: Information Content.

**Table 4:** Reporter occupations for FAERS reports of PPI-associated AKI and TIN, 2020–2025.

Occupation	Acute Kidney Injury	
	n	%
Nurse	4627	33.9
Consumer	2062	15.1
Healthcare Professional	864	6.3
Physician	1018	7.5
Pharmacist	236	1.7
Not Specified	4847	35.5
	**Tubulointerstitial Nephritis**	
Nurse	602	25.0
Physician	378	15.7
Healthcare Professional	351	14.6
Consumer	280	11.6
Pharmacist	93	3.9
Not Specified	705	29.3

**Table 5:** Clinical outcomes of FAERS reports of PPI-associated AKI and TIN, 2020–2025.

Outcome	Acute Kidney Injury	
	n	%
Hospitalized	1685	12.3
Death	1240	9.1
Disability	157	1.1
Life-threatening	90	0.7
Congenital Anomaly	5	0.04
Required Intervention	1	0.01
Other	10,332	75.7
Not Specified	144	1.1
	**Tubulointerstitial Nephritis**	
Hospitalized	546	22.7
Death	120	5.0
Disability	41	1.7
Life-threatening	27	1.1
Congenital Anomaly	1	0.04
Required Intervention	1	0.04
Other	1600	66.4
Not Specified	73	3.0

## Data Availability

The data analyzed in this study are publicly available from the FDA Adverse Event Reporting System (FAERS) Public Dashboard: https://www.fda.gov/drugs/fdas-adverse-event-reporting-system-faers/fda-adverse-event-reporting-system-faers-public-dashboard (accessed on 3 February 2026).

## Supplementary Materials

The following supporting information can be downloaded at: https://www.mdpi.com/article/10.3390/jcm15031298/s1, Table S1: Agent-stratified descriptive composition of AKI reports within FAERS for individual proton pump inhibitors, by year (2020–2025); Table S2: Agent-stratified descriptive composition of tubulointerstitial nephritis (TIN) reports within FAERS for individual proton pump inhibitors, by year (2020–2025).
