# Supplementary material for: Proton Pump Inhibitors and Disproportionate Reporting of Acute Kidney Injury and Tubulointerstitial Nephritis: A FAERS Pharmacovigilance Study, 2020–2025
Source: J Clin Med. 2026 Feb 6;15(3):1298. doi: 10.3390/jcm15031298 (PMC12897620; doi:10.3390/jcm15031298)
Supplement: Supplementary file 1 [file jcm-15-01298-s001.zip › jcm-4101330-supplementary.pdf]

Supplementary Table S1. Agent-stratified descriptive composition of AKI reports within FAERS for individual proton pump inhibitors, by year (2020–2025). Values are shown as the number of AKI reports in which the PPI was reported (numerator) divided by the total FAERS reports for that PPI in the same year (denominator).

AKI reporting composition within FAERS (n/total FAERS reports for that PPI in the same year, %)	2020	2021	2022	2023	2024	2025
Omeprazole	842/4867 (9.9%)	695/3788 (18.34%)	342/2710 (12.6%)	198/2489 (7.95%)	76/1718 (4.42%)	34/839 (4.05%)
Esomeprazole	2123/6279 (33%)	1826/4905 (37.22%)	827/2507 (32.98%)	392/1668 (23.5%)	97/1588 (6.1%)	30/1275 (2.35%)
Lansoprazole	1121/3643 (30%)	1181/3319 (35.58%)	532/1928 (27.59%)	181/1307 (13.8%)	43/1037 (4.14%)	25/553 (4.52%)
Pantoprazole	906/4679 (19.36%)	896/3900 (22.97%)	621/3190 (19.46%)	118/2080 (5.6%)	90/2105 (4.27%)	52/928 (5.6%)
Rabeprazole	9/196 (4.59%)	2/201 (0.99%)	2/184 (1.08%)	0/248 (0%)	4/214 (1.86%)	1/63 (1.58%)
Dexlansoprazole	142/669 (21.22%)	93/577 (16.12%)	118/597 (19.76%)	31/321 (9.65%)	3/208 (1.4%)	1/77 (1.2%)

These proportions describe reporting composition within FAERS and should not be interpreted as incidence, absolute risk, or comparative safety across PPI agents, because FAERS lacks exposure denominators and reporting is influenced by utilisation, channeling, and time-varying reporting behaviour.

Supplementary Table S2. Agent-stratified descriptive composition of tubulointerstitial nephritis (TIN) reports within FAERS for individual proton pump inhibitors, by year (2020–2025). Values are shown as the number of TIN reports in which the PPI was reported (numerator) divided by the total FAERS reports for that PPI in the same year (denominator).

TIN reporting composition within FAERS (n/total FAERS reports for that PPI in the same year, %)	2020	2021	2022	2023	2024	2025
Omeprazole	215/4867 (4.42%)	155/3788 (4.10%)	69/2710 (2.55%)	58/2489 (2.33%)	62/1718 (3.61%)	25/839 (2.98%)
Esomeprazole	335/6279 (5.34%)	274/4905 (5.59%)	145/2507 (5.78%)	88/1668 (5.28%)	21/1588 (1.32%)	22/1275 (1.73%)
Lansoprazole	168/3643 (4.61%)	144/3319 (4.34%)	84/1928 (4.36%)	28/1307 (2.14%)	6/1037 (0.60%)	4/553 (0.72%)
Pantoprazole	132/4679 (2.82%)	107/3900 (2.74%)	77/3190 (2.41%)	42/2080 (2.02%)	54/2105 (2.57%)	19/928 (2.05%)
Rabeprazole	12/196 (6.12%)	4/201 (2.00%)	7/184 (3.80%)	5/248 (2.02%)	2/214 (0.93%)	3/63 (4.76%)
Dexlansoprazole	16/669 (2.40%)	13/577 (2.25%)	9/597 (1.51%)	4/321 (1.25%)	0/208 (0%)	0/77 (0%)

These proportions describe reporting composition within FAERS and should not be interpreted as incidence, absolute risk, or comparative safety across PPI agents, because FAERS lacks exposure denominators and reporting is influenced by utilisation, channeling, and time-varying reporting behaviour.
